# Trigeminous Reflexes

**Published:** 1898-06

**Authors:** H. H. Johnson

**Affiliations:** Macon, Ga.


### Trigeminous Reflexes.

BY H. H. JOHNSON, D.D.S., MACON, GA.

Abstract of paper read at St. Augustine, Florida.

The trigemini is a source of common interest to the dentist and the ophthalmologist. From the variety of its functions and the complexity of its connections and distributions, reflexes difficult of diagnosis are more frequently met with in its territory than in any other. A thorough knowledge of its anatomy, with its physiological and pathological manifestations is exceedingly desirable in order that we may accurately diagnose the many perplexing cases which come before us. Pathological reflexes are most frequently met with in the terminal branches of the dental division, as odontalgia manifested in a superior tooth when its origin is irritation of an inferior one, or the irritation of a tooth may manifest itself in the eye, the ear, the nose, or in general neuralgic symptoms, without reference to the real seat of trouble. An operation upon the nasal septum will produce neuralgia in the superior anterior teeth. A case was cited in his own practice of severe neuralgia in the region of the left temple. Exploration of a cavity in the posterior proximal surface of the second superior left upper molar, called forth the exclamation—"That felt as if you had driven a nail into my brain," but no pain was felt in the tooth though the exposed nerve had been touched by the probe. After the nerve was killed and the tooth filled there was no more neuralgia in the temple. In another case months of suffering from chronic neuralgia, but with no trouble with the teeth, was cured by the removal of the pulps of the lower third molars, both of which contained pulp nodules. Another case of neuralgic suffering for several years showed no dental trouble, except some recession of the gum around the first upper molar. The tooth was opened into and a layer of secondary dentine found covering almost the entire floor of the chamber, nearly cutting off communication between the body of the pulp and the tissue in the canals. Removing the pulp and filling the tooth gave entire relief.

Dr. Johnson cited a case from Dr. Brubaker in which severe toothache followed a surgical operation in the nasal cavity; one from Dr. Hutchinson of pain in the teeth and ear coincident with ulceration of the cornea; from Dr. W. H. Morgan of the cure of a raving maniac by the extraction of an offending tooth. Cases of insanity caused by dental irritation, reported in the American System of Dentistry, were also referred to, all tending to show the serious importance of the subject.

In concluding his paper, Dr. Johnson spoke of the efforts made in his State to secure the appointment of dental surgeons in insane asylums, which he considered of even greater importance than their appointment in the army and navy.

DISCUSSION.

In the discussion of this paper, Dr. Crawford said that wherever a large number of inmates were confined within the limits of a State institution, the Commonwealth that failed to provide for the care of the teeth of such inmates as carefully as for other parts of the organism failed in the performance of its duty towards those unfortunates. Dr. Crawford spoke of the tendency of modern medicine towards ignoring old doctrines, as of idiopathy, reflex action, as not supported by the voice of science—but, he said, the voice of God Almighty says it is so; and that is on our side. The vast field of nervous reflex action can not be taken away from dental surgery. It must be borne in mind, however, that the phenomena of reflex action rarely occur in connection with dead teeth, after the destruction of the pulp.

Dr. Walker expressed surprise that Dr. Crawford should except the eyeball from the effects of reflex neuroses from the trigeminores, and described the distribution of the ciliary and other nerves, as illustrated in the latest edition of Gray's Anatomy.

Dr. Chapple repeated the statement of Prof. Garretson that nine-tenths of all neuralgias and other diseases of the human head were due to diseased teeth and that the eye made no exception, and cited cases in illustration.

Dr. B. Holly Smith, and Dr. Frank Woodbury, of Halifax, N. S., and others cited cases of insanity and other grave troubles cured by attention to the teeth.

Dr. E. P. Beadles attributed the remarkable indifference to this very interesting subject to the fact that the medical profession, as a rule, attach so little importance to the teeth, apparently regarding them as organs which have nothing whatever to do with the rest of the organization.

Dr. T. P. Hinman expressed the view that tissues which are associated in their development are also associated in diseased conditions. Thus the fact that the teeth and the internal ear being both developed from an involution of the epiblastic layer, explains the connection between diseased teeth and the earaches and abscesses of the ear, of which he cited cases.

